# Community-based surveillance contribution to the response of COVID-19 in Niger

**DOI:** 10.11604/pamj.2021.40.88.28175

**Published:** 2021-10-11

**Authors:** Ahmed Abani Maazou, Batouré Oumarou, Baruani Bienvenu, Blanche-Philomene Melanga Anya, Tambwe Didier, El Khalef Ishagh, Biey Joseph Nsiari-muzeyi, Patrick Katoto, Charles Shey Wiysonge

**Affiliations:** 1 Country Office, World Health Organization, Quartier Plateau, Avenue Mohamed VI 1204, Niamey, Niger,; 2 Sub-Regional Office for West Africa, World Health Organization, Independence Street, Gate 0058, Ouagadougou, Burkina Faso,; 3 Centre for Infectious Diseases, Faculty of Medicine and Health Sciences, Stellenbosch University, Francie Van Zijl Drive, Tygerberg 7505, Cape Town, South Africa,; 4 Centre for Tropical Medicine and Global Health, Faculty of Medicine, Catholic University of Bukavu, Bugabo 02, Bukavu, Democratic Republic of Congo,; 5 Department of Global Health, Faculty of Medicine and Health Sciences, Stellenbosch University, Francie van Zijl Drive, Tygerberg 7505, Cape Town, South Africa,; 6 Cochrane South Africa, South African Medical Research Council, Francie Van Zijl Drive, Parow Valley 7501, Cape Town, South Africa,; 7 School of Public Health and Family Medicine, University of Cape Town, Anzio Road, Observatory 7935, Cape Town, South Africa

**Keywords:** Integrated disease surveillance and response, people centered-surveillance, case-findings, SARS-CoV-2

## Abstract

**Introduction:**

the COVID-19 pandemic has spread across all countries in Africa, with much of the model forecasting disastrous results owing to weak health services and political uncertainty. In Niger, an adaptive solution to the COVID-19 pandemic has been implemented by community-based surveillance system (CBS) to complement passive case-finding in health systems.

**Methods:**

the CBS program was designed to use the current CBS polio network spanning 37 health districts in six regions. Between April and November 2020, 150 community health workers (CHWs) were equipped to improve integrated disease surveillance and response (IDSR) preparedness and response to the COVID-19 pandemic. We retrospectively analysed the health data of the National Health Information System to describe the effect of CBS in COVID-19 surveillance.

**Results:**

overall, trained CHWs were able to raise awareness among 2,681,642 persons regarding COVID-19 preventions and controls strategies. They reported 143 (84%) valid alerts resulting in two positive COVID-19 cases missing in the community. In addition, CHWs added to the contact tracing of 37 individuals and informed about the deaths in the community.

**Conclusion:**

community-based surveillance improved COVID-19 response in Niger. Logistic assistance and ongoing training are the foundations for increasing and sustaining the sensitivity of CBS systems in response to the COVID-19 pandemic to deter hotspots across countries.

## Introduction

The World Health Organization (WHO) was alerted on 31^st^ December 2019 to the outbreak of atypical pneumonia in Wuhan, China [1]. On 30^th^ January 2020, the WHO triggered the highest level of warning by announcing a public health emergency of international significance following human-to-human transmission outside China to COVID-19 [2]. On 11^th^ March 2020, profoundly alarmed at both the unprecedented levels of dissemination and the seriousness of the outbreak, the WHO referred to COVID-19 as a pandemic and thereby becoming the first pandemic caused by coronavirus [3]. The African region, like other continents, is also affected by the pandemic: as of November 25, 2020, 47 countries were affected with 1,451,296 cumulative cases, including 24,454 deaths [4].

Niger, one of the West African nations, reported the first case of COVID-19 in Niamey (capital city) on 19 March 2020. As of November 25, 2020, there were 1,381 confirmed cases including 70 deaths [5]. The response to the pandemic was coordinated in the sense of a shortage of human capital in health facilities, especially in integrated health centers. Surveillance is largely passive by health institution [6], i.e. in the basis of the voluntary use of health care by the public. Active case research is not carried out routinely, on the one hand, by health staff, but rather by the epidemiological monitoring mechanism developed in the sense of disease control. However, a low insurance coverage (49%) does not provide access to health care for the whole population, which prevents the identification of the highest number of patients by health facilities.

Community-based surveillance (CBS) is a coordinated and quick compilation of community information, usually signs of diseases that may pose a possible danger to public health [6]. In the case of COVID-19, an aggressive search is made for subjects with disease warning signs/symptoms that need to be found at the group level, including contacts with known reported cases. Niger has a CBS program, including polio surveillance in 13 health districts in the regions of Diffa (6), Zinder (2), Maradi (1) and Tillabéri (4). We anticipated that an effective CBS can, among other aspects, strengthen links between populations and their local health system and integrate an active rather than passive monitoring system. Eventually, a working CBS would support both the communities and the broader health care sector. The method is based on volunteer Community Health Workers (CHWs) with different background using a generalized case description to scan for and submit alerts to health workers.

## Methods

**Study design, setting and population:** we report a descriptive work conducted between April and November 2020 among 2,150 CHWs selected to train for CBS to strengthen the integrated disease surveillance and response (IDSR) preparedness and response against COVID-19 pandemic. The work was conducted across 37 health districts throughout six regions in Niger. The health district was included if it has notified at least one case of COVID-19 infection and/or if it was geographically located near a country that has reported COVID-19 cases (e.g. neighbouring Nigeria where trade is carried out daily). At the selected health district, the recruited HCW was a permanent resident of the village, with some schooling (at least at the elementary level and able of, reading and writing), accessible and ready to volunteer. Non-residents, people under the age of 18 and those without formal education were consequently disqualified. Gradual training was organized for CHWs. Training was based on the WHO guidance including 1/orientation on the recognition of COVID-19, 2/public health incidents for use at community level, based on the nationally adapted IDSR Guideline-IDSR diseases, and 3/ developing a CBS structure-customer-based surveillance monitoring and response.

**Data collection procedures and variables definition:** every CHW was assigned a village of intervention and carried out home visits, to raise awareness on infection, prevention, and control (IPC) of COVID-19, and to alert suspicious cases. Intervention was also applied to ceremonies including mass gatherings such as weddings, baptisms, funerals, etc. The appraisal was accompanied by the sending of a daily notice, which was also followed by a weekly compilation report. Data was collected by tools built for this purpose. This included data collection media/tally sheet (scorecard and weekly report sheet) made available to CHWs to allow them to collect and transmit information from their villages to the primary health centre or the associated integrated health centre. Finally, an Excel form was created for the centralization of data by the district epidemiologist in charge of surveillance. From the district level, the circuit follows the Regional Directorate of Public Health (DRSP) and then the Central Supervisory Directorate in accordance with the alert management scheme. To determine the contribution of CHW, the key variables chosen included number of persons who received the information, alerts issued, alerts confirmed, suspected cases examined, suspected cases sampled, positive COVID-19 cases, contacts established, contacts followed, community deaths registered, community deaths investigated and sampled, and franchises visited. An alert was judged to be valid if it met the community definition of COVID-19 or an asymptomatic subject wishing to perform a COVID-19 test either because he has been in contact with a positive case or wished to plan a trip; the subject become suspicious if he presented the aforementioned conditions and/or clinical signs in favour of COVID-19 such as cough and/or fever with one or more of the following signs: headaches, myalgia, poly arthralgia, diarrhoea, runny nose, anaemia, ageusia, fatigue, etc..). The suspect case was then sampled once informed consent was obtained.

**Data analysis:** we used Excel spreadsheet (Microsoft Office 2016) to follow the pattern on a regular basis and to summarize the data as frequency/proportion. GraphPad prism v9.0 was used to create visual graphics.

**Ethical approval:** we report information collected as part of a public health programme, which does not require ethical approval. We have removed all identifiable information from the data reported in this article.

## Results

**Figure 1:**
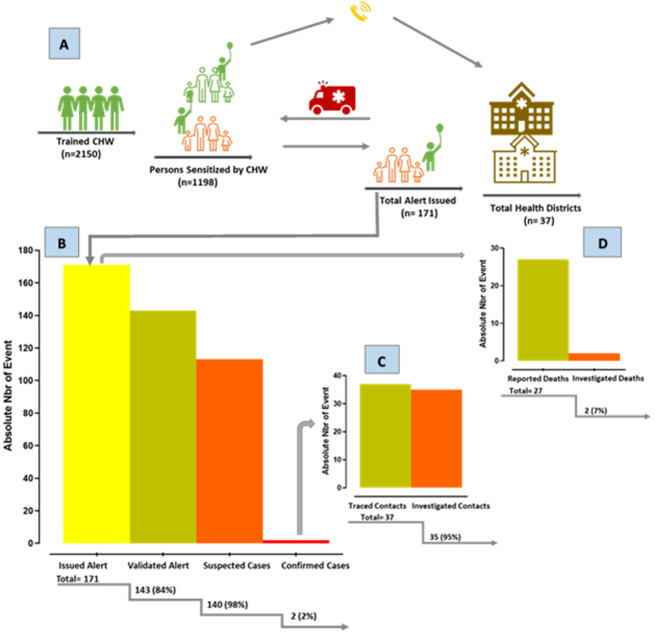
(A) designing community-based surveillance; (B) for active COVID-19 case-finding; (C) contact-tracing and death-report; (D) across 45 health districts in Niger, April-November 2020

**Population awareness:** In Figure 1, panel A, demonstrates the process followed by CHWs in effort to implement CBS in relation to COVID-19 in Niger. Overall, 37 health districts spanning six regions (Agadez, Diffa, Dosso, Maradi, Tahoua and Zinder) accounting for a population of 13,810,910, were visited. With a total of 2,150 CHWs trained, 2,681,642 persons received the information with regard to the COVID-19 public health threat and related basic IPC with most of them being in Zinder (80%) and the least in Tahoua (1%) (Table 1). At the personal level, each CHW raised an average of 1,198 individuals, ranging from 94 in Tahoua to 2,310 in Zinder.

**Table 1:** key indicators of community-based surveillance for active COVID-19 case-finding, contact-tracing, and death-report by region in Niger, April-November 2020

Absolute Number of	Regions						
	Agadez	Diffa	Dosso	Maradi	Tahoua	Zinder	Total
**Trained CHW**	65 (3.0)	230 (10.7)	571 (26.6)	198 (9.2)	170 (7.9)	916 (42.6)	2150 (100.0)
**Persons who received the information**	105,711 (3.9)	10,9973 (4.1)	42,722 (1.6)	291,129 (10.9)	16,019 (0.6)	2,116,088 (78.9)	2,681,642 (100.0)
**Alerts issued by CHW**	17 (9.9)	16 (9.4)	126 (73.7)	10 (5.8)	15 (8.8)	4 (2.3)	171 (100.0)
**Alerts validated**	17 (11.9)	0 (0.0)	106 (74.1)	2 (1.4)	15 (10.5)	3 (2.1)	143 (100.0)
**Alert number/100,000 inhab**	2.72	5.27	7.95	0.41	0.37	0.08	1.36
**Suspected cases investigated**	17 (12.1)	0 (0.0)	106 (75.7)	1 (0.7)	13 (9.3)	3 (2.1)	140 (100.0)
**Suspected cases sampled**	1 (0.9)	0 (0.0)	106 (93.8)	0 (0.0)	5 (4.4)	1 (0.9)	113 (100.0)
**Positive cases COVID-19**	1 (50.0)	0 (0.0)	1 (50.0)	0 (0.0)	0 (0.0)	0 (0.0)	2 (100.0)
**Case contact identified**	6 (16.2)	0 (0.0)	12 (32.4)	0 (0.0)	11 (29.7)	8 (21.6)	37 (100.0)
**Case contacts followed**	6 (17.1)	0 (0.0)	12 (34.3)	0 (0.0)	11 (31.4)	6 (17.1)	35 (100.0)
**Community deaths investigated and sampled**	0 (0.0)	0 (0.0)	0 (0.0)	2 (100.0)	0 (0.0)	0 (0.0)	2 (100.0)
**Positive community deaths**	0 (0.0)	0 (0.0)	0 (0.0)	0 (0.0)	0 (0.0)	0 (0.0)	0 (0.0)

Note: data between brackets are percent. Abbreviation: CHW: community health workers; Inhab: inhabitant

**Active case findings:** In Figure 1, Panel B displays case-findings outcomes by CHWs for COVID-19 in general while Table 1 shows records by regions. Of the 171-alert issued, 143 (84%) have been validated by the district team representing 1.36 valid alert/ 100000 inhabitants. Most alerts came from Dosso (74%), followed by Agadez (10%), Diffa and Tahoua (9%), Maradi (6) and Zinder (2%). The ratio of valid alerts/ alerts issued showed a huge variation across regions with the perfect match found in Agadez and Tahoua (100%), following with Dosso (84%), Zinder (75%) while only 20% was validated in Maradi and none in Diffa. Of the 143 (84%) valid alerts, 140 (98%) have been declared as suspected cases. Of these, three-quarter came from Dosso. The ratio suspected case/valid alert highlighted a perfect match in Agadez and Dosso (100%), then in Tahoua (87%) and Maradi (50%). While only 113 (80%) of suspected cases have been sampled for COVID-19 RT-PCR testing, all 106 cases from Dosso have been sampled, 5/13 in Tahoua, 1/3 in Zinder, 1/17 in Agadez and 0/1 in Maradi. Further, the only case sampled in Agadez was found to be positive for COVID-19 whilst of the 106 cases sampled in Dosso, only one was confirmed a COVID-19 case.

**Contacts tracing and mortality report:** Figure 1, Panels C and D present contacts tracing, and mortality report outcomes respectively issued by CHWs for COVID-19 in general while Table 1 shows records by regions. Overall, 37 contacts have been identified by CHWs with half of them located in the same region where community confirmed COVID-19 have been reported by CHWs (Agadez and Dosso) and all were investigated. Other reported contacts were from Tahoua (31%) and Zinder (17%) yielding a total of 35 contacts investigated. Finally, of the 27-death occurred in the community during the study period, only 2 (7%) were suspect for COVID-19. They were both reported in Maradi and were investigated, but none of them was confirmed by RT-PCR as infected by SARS-CoV-2 infection.

## Discussion

For the first time, we have reported and documented Integrated Disease Surveillance and Response (IDSR) using Community-based Surveillance Preparedness and Response to the Niger Pandemic of COVID-19. The CBS program has successfully educated 2,150 CHWs who have increased awareness of the COVID-19 pandemic among 2.6 million people across six Nigerian regions. A functioning structure led by the Ministry of Health has been successfully developed and has made it possible to link CHWs at the community level to their primary health centre which connects to the integrated health centre and, subsequently, to the district level. This system has delivered 143 timeline alerts sent by CHWs to be validated, 140 suspected cases to be quarantined and 113 suspected cases to be accurately sampled to confirm SARS-CoV-2 infection.

Our results have suggested a large difference in the outcomes of the CBS. We trained more CHWs in Dosso and Zinder and received most of the warnings in the provinces of Dosso and Agadez. The ratio of credible alerts to warnings issued revealed a significant variance across regions, with a complete fit between Agadez and Tahoua relative to Dosso and Zinder with a wide disparity in other regions. The suspected case-valid warning ratio, however, illustrated the ideal fit between Agadez and Dosso following Tahoua. Sub-consequently, the total ratio of valid alarms/warnings issued was poor (83%) relative to the suspicious case/valid alarm ratio (98%) suggesting that continued training is needed to further increase the quality of alerts issued. Similarly, provided that only 80% of suspicious cases were sampled, persistent discussion is invited to aid in the collection of cases to be sampled. As a result, we found that the only case sampled in Agadez was determined to be positive for COVID-19, although only one of the 106 cases sampled in Dosso was confirmed for COVID-19. This is also significant, as the issue posed is whether precision and sensitivity can be improved while responding to a global pandemic. While there is a great need for sensitivity to urgently recognize the danger of COVID-19 contamination at the population level, for effective public health interventions (e.g. quarantine, contact tracing) to be implemented, concomitantly, the specificity of RT-PCR testing capability in many remote areas should be increased.

Contact tracing tends to be a cornerstone in restricting the spreading of COVID-19 at the population level. Since clinical and laboratory-based monitoring is passive (identifying disease cases only among those present for treatment), it must be complemented by strategies to recognize lost cases, validate diagnosis, and investigate community-based outbreaks [7]. Our model has been able to successfully monitor and quarantine 35 and 37 contacts respectively, half of which were in the area where community cases were reported. In designing a reporting policy, policymakers would need to strike an acceptable balance between sensitivity (identifying all big events) and sustainability (maintaining reporting power without sacrificing other public health functions). Governments should ensure that incident evaluation teams responding to community reports have the appropriate powers to conduct event-based monitoring in community environments and where possible, to enforce immediate public health initiatives.

The introduction of the CBS in the COVID-19 pandemic response in Niger has necessitated a close partnership between the state of Niger, a network of collaborators and various stakeholders at both community and district levels. The discrepancies found in the CBS outcomes by region is common for CBS in low and middle-income countries [8, 9] and in our case, it may be explained by the progressive standard of training following regional deployment and logistical problems. Moreover, the denial of the disease by the population (white invention) especially in the regions of Zinder and Maradi which are also the most populated and bordering Nigeria might explain such poor performance. Incentives were not adequately addressed in this work but have been recognized as a key obstacle to implementation across agencies as financial and material benefits are most likely to affect CHW's ownership of the project and in turn, the timely gathering of data and the longevity of the programme. Studies have challenged the viability of CBS outside extreme emergencies and outbreaks due to the need for additional funding from non-governmental agencies and the increasing obligations of CHWs [9]. By building a CBS system on the polio legacy, we offer a model that might help other countries to overcome structural challenges paused by establishing a *de novo* CBS to respond to an emerging public health crisis.

## Conclusion

The COVID-19 pandemic has surged in an already stretched health system in Africa. We demonstrated the feasibility of a community-based surveillance (CBS) system built on existing polio vaccination infrastructure, instead of implementing a *de novo* CBS system at the beginning of the pandemic. This has contributed to strengthen pandemic surveillance, particularly at the community level. Whenever feasible, efforts should be invested to improve case tracking and retro-feedback mechanisms between community networks and health facilities to sustain the national effort response to the COVID-19 epidemic.

### What is known about this topic

- Community based surveillance is a key strategy to support integrated care;
- Quality data and contact-tracing are important to capitalize states´ efforts in containing the spread of COVID-19 pandemic.

### What this study adds

- In low-income settings, Community-based surveillance can help improve COVID-19 case findings;
- Using the poliomyelitis fighting experience to strengthen surveillance at the community level can help fighting COVID-19 pandemic in Africa.
